# Effectiveness and safety of regimens containing linezolid for treatment of *Mycobacterium abscessus* pulmonary Disease

**DOI:** 10.1186/s12941-023-00655-2

**Published:** 2023-12-06

**Authors:** Li-ping Cheng, Qing Zhang, Hai Lou, Xiao-na Shen, Qing-rong Qu, Jie Cao, Wei Wei, Wei Sha, Qin Sun

**Affiliations:** Clinical and Research Center for Tuberculosis, Shanghai Key Laboratory of Tuberculosis, Shanghai Pulmonary Hospital, School of Medicine, Tongji University, Shanghai, 200433 China

**Keywords:** *Mycobacterium abscessus*, Linezolid, Pulmonary Disease, Treatment

## Abstract

**Objective:**

To evaluate the effectiveness and safety of linezolid-containing regimens for treatment of *M. abscessus* pulmonary disease.

**Methods:**

The records of 336 patients with *M. abscessus* pulmonary disease who were admitted to Shanghai Pulmonary Hospital from January 2018 to December 2020 were retrospectively analyzed. A total of 164 patients received a linezolid-containing regimen and 172 controls did not. The effectiveness, safety, antibiotic susceptibility profiles, outcomes, culture conversion, cavity closure, and adverse reactions were compared in these two groups.

**Results:**

The two groups had similar treatment success (56.1% vs. 48.8%; *P* > 0.05), but treatment duration was shorter in the linezolid group (16.0 months [inter-quartile ranges, IQR: 15.0–17.0] vs. 18.0 months [IQR: 16.0–18.0]; *P* < 0.01). The rates of sputum culture conversion were similar (53.7% vs. 46.5%, *P* > 0.05), but time to conversion was shorter in the linezolid group (3.5 months [IQR: 2.5–4.4] vs. 5.5 months [IQR: 4.0–6.8]; *P* < 0.01). The linezolid group had a higher rate of cavity closure (55.2% vs. 28.6%, *P* < 0.05) and a shorter time to cavity closure (3.5 months [IQR: 2.5–4.4] vs. 5.5 months [IQR: 4.0–6.8]; *P* < 0.01). Anemia and peripheral neuropathy were more common in the linezolid group (17.7% vs. 1.7%, *P* < 0.01; 12.8% vs. 0.6%, *P* < 0.01).

**Conclusions:**

The linezolid and control groups had similar treatment success rates. The linezolid group had a shorter treatment duration, shorter time to sputum culture conversion, and higher rate and shorter time to lung cavity closure. More patients receiving linezolid developed anemia and peripheral neuropathy.

**Supplementary Information:**

The online version contains supplementary material available at 10.1186/s12941-023-00655-2.

## Introduction

The incidence of infections with non-tuberculous mycobacteria (NTM) has increased during recent decades, and these infections are now a threat to public health worldwide [1–3]. *Mycobacterium abscessus* is one of the most common causes of NTM disease, and is the second most frequently isolated NTM species in many regions of China (after *M. avium*) [4–6]. Due to the high frequency of mutations and acquired drug resistance [7, 8], treatment of *M. abscessus* pulmonary disease (MAB-PD) requires long-term administration of a combination of drugs [9]. Unfortunately, outcomes of treatment remain unsatisfactory [10], and there is a high rate of recurrence [11]. Our previous large retrospective study found that *M. abscessus* was the second most common cause of NTM disease, and accounted for 29% of all isolates in Shanghai from 2014 to 2018 [6]. We also found that treatment failures were more likely for MAB-PD than for infections by *M. avium* and *M. kansasii* [6].

Linezolid is a synthetic antibacterial agent in the oxazolidinone family that has bactericidal activity against most fast-growing mycobacteria. The 2016–2021 WHO guidelines consider linezolid one of the core drugs for treatment of multidrug-resistant tuberculosis (MDR-TB) [12]. Linezolid inhibits the translation of mRNA by binding to the 50 S subunit of the bacterial ribosome, thereby preventing formation of the 70 S initiation complex [13, 14]. The susceptibility of clinical isolates of *M. abscessus* to linezolid varies from 29 to 76.9%, depending on the geographical region [15, 16]. In addition, linezolid exhibits in vitro synergistic effects with moxifloxacin and cefoxitin against *M. abscessus* [17].

The official ATS/IDSA guideline of 2007 suggested that treatment of MAB-PD should be based on the results of antibiotic susceptibility testing, and should consist of multidrug therapy that includes macrolides and one or more parenteral agents (amikacin, cefoxitin, or imipenem) [18]. A statement by the ATS/ERS/ESCMID/IDSA in 2020 recommended linezolid treatment for the initial and subsequent stages of MAB-PD [19]. However, clinical data on linezolid for treatment of MAB-PD is very limited. There is no summary of data from large studies, there are few prospective clinical studies, and most studies only examined a small number of cases. Hence, the current evidence supporting use of linezolid for treatment of MAB-PD is limited.

We performed a retrospective study in a relatively large sample of patients who were diagnosed with MAB-PD at our center. The aim was to evaluate the clinical effectiveness and safety of linezolid-containing regimens for treatment of MAB-PD.

## Materials and methods

### Study subjects

Between January 1, 2018 and December 31, 2020, the records of all patients who were admitted to the Shanghai Pulmonary Hospital and diagnosed with MAB-PD were examined. This study was approved by the Ethics Committees of Shanghai Pulmonary Hospital (Ethics No. fk22-005). The inclusion criteria were age 18 years or older, diagnosis of MAB-PD, completion of treatment as prescribed and follow-up for at least 1 year after drug withdrawal, HIV negativity, and receipt of linezolid treatment for more than 12 months (linezolid group only). The exclusion criteria were pregnancy or breastfeeding, pulmonary tuberculosis or co-infection with another NTM species, active autoimmune disease requiring corticosteroid or immunosuppressant agents, advanced stage of malignancy, and incomplete clinical data. All patients who received linezolid were included in the linezolid group, and patients who received therapy that did not include linezolid were in the control group.

The diagnosis of MAB-PD was based on a previous consensus report (“An official ATS/ IDSA Statement: Diagnosis, Treatment, and Prevention of Nontuberculous Mycobacterial Diseases”). *M. abscessus* was isolated from at least two separate expectorated sputum samples or at least one bronchoalveolar lavage fluid (BALF) sample [18].

### Species identification and drug susceptibility tests

Mycobacterial cultures were performed using mycobacterial growth indicator tubes (BACTEC MGIT 960 System, Becton Dickinson Life Sciences) according to the manufacturer’s instructions. Briefly, a 1 mL treated sample was added into an MGIT culture tube with 0.8 mL additives, and incubated for 6 weeks. The NTM isolates were then confirmed using MPB64 detection, and the p-nitrobenzoic acid and thiophene-2-carboxylic acid hydrazine tests.

Identification of NTM isolates was performed by PCR reverse dot-blot hybridization (*Mycobacterium* species identification gene detection kit, Shanghai Xin Peijing Medical Inspection Center, batch No.201,707,002) and matrix assisted laser desorption ionization time-of-flight mass spectrometry (MALDI-TOF MS; Shanghai Kangli Medical Inspection Institute ® System).

Antibiotic susceptibility was tested according to the M24-A2 guidelines issued by the American Clinical and Laboratory Standards Institute (CLSI). The minimum inhibitory concentration (MIC) values of 11 antibiotics against *M. abscessus* (amikacin, cefoxitin, ciprofloxacin, clarithromycin, doxycycline, imipenem, linezolid, minocycline, moxifloxacin, tobramycin, and sulfamethoxazole) were determined by the broth microdilution method (Sensititre, Thermo Scientific). All results were interpreted using the criteria recommended in the CLSI M24-A2 guideline.

### Treatment regimens

Treatments were determined by the antibiotic susceptibility results, the guideline of the British Thoracic Society [1], and the 2012 Expert Consensus for the Diagnosis and Treatment of Non-tuberculous Mycobacteria Disease from the Chinese Medical Association. For the initial phase, at least three of the following drugs were used: amikacin (intravenous, 10–15 mg/kg/day), clarithromycin (oral, 1000 mg/day) or azithromycin (oral, 500 mg/day), imipenem (intravenous, 2000 mg/day) or cefoxitin (intravenous, 6–12 g/day), linezolid (oral, 600 mg/day), doxycycline (oral, 200 mg/day), clofazimine (oral, 100 mg/day), and moxifloxacin (oral, 400 mg/day). During the continuation phase, at least two of the following drugs were used: amikacin (400 mg/day, nebulised), clarithromycin or azithromycin (doses as above), linezolid (dose as above), doxycycline (dose as above), and clofazimine and moxifloxacin (doses as above). In all patients, treatment was given for at least one year after sputum culture conversion or for 2 years.

### Study design and follow-up

Patients were followed from initiation of anti-NTM therapy to death or censoring from loss to follow-up or the decision to terminate treatment. The demographic characteristics, clinical symptoms, laboratory results, radiological features, antibiotic susceptibility test results, outcomes, and adverse effects and events were recorded. Each patient was followed up in an outpatient clinic or an inpatient department after discharge.

The baseline characteristics of enrolled patients were recorded, including the results of NTM cultures, antibiotic susceptibility tests, and chest computed tomography (CT), all of which were performed within 1 month before the start of antibiotic therapy. During therapy, sputum culture was performed at least once every 2 months and chest CT examination every 3 months. To monitor adverse effects and events during therapy, an electrocardiogram and measurements of whole blood cell count, blood chemistry (indicators of liver and kidney function), and electrolytes were performed monthly. Patients on amikacin were screened for hearing loss every 3 months or when indicated. Patients in the linezolid group had a routine blood test every 2 weeks during the first 3 months of treatment. All patients were encouraged to report all adverse events (such as nausea, vomiting, appetite loss, vision loss, and hearing loss) to their physicians during the follow-up period, and symptomatic treatment was offered following corresponding guidelines.

### Treatment outcomes

Culture conversion was defined as the finding of at least three consecutive negative mycobacterial cultures from sputum collected at least 4 weeks apart during antimycobacterial treatment. The sample date of the first negative culture was considered the date of culture conversion.

Treatment effectiveness [20] was defined as microbiological cure (finding multiple consecutive negative but no positive cultures with the causative species from sputum after culture conversion and until the end of antimycobacterial treatment) or as microbiological failure (re-emergence of multiple positive cultures or persistence of positive cultures with the causative species from sputum after 12  or more months of antimycobacterial treatment while the patient was still receiving treatment). In this study, treatment success was defined by microbiological cure, and all other outcomes were defined as treatment failure.

Chest CT was performed using 64-slice examinations (1-mm section thickness) and the results were independently evaluated by 2 radiologists and 1 infectious diseases physician. Any discrepancy was resolved by their discussion. The maximum diameter of a measured cavity was used to determine cavity changes (closure, smaller, no change, larger or new cavities), and changes in shape, location, and size were evaluated for all lesions (absorption, no change, or progression).

All patients received anti-NTM treatment until at least 1 year after the sputum culture conversion or up to 2 years if cultures did not turn negative.

### Statistical analysis

Statistical analyses were performed using SPSS version 20.0 (SPSS Inc., Chicago, IL, USA). Continuous variables with normal distributions are presented as means ± standard deviations and compared using Student’s *t*-test. Continuous variables with non-normal distributions are presented as medians ± inter-quartile ranges and compared using the *Mann-Whitney U* test. Categorical variables are reported as frequencies (percentages) and compared using the Chi-square test or using Fisher’s exact test (when the theoretical frequency was less than 5). A *P* value below 0.05 in a two-sided test was considered to indicate statistical significance. Kaplan-Meier curves were plotted to compare the two groups in terms of sputum culture conversion and cavity closure, and the statistical significance of differences was determined using the log-rank test. Unconditional binary logistic regression (forward conditional method) was used to identify risk factors for treatment failure, and adjusted odds ratios (aORs) and 95% CIs were then calculated for each antibiotic.

## Results

### Demographics, clinical characteristics, and prior treatments

**Fig. 1:**
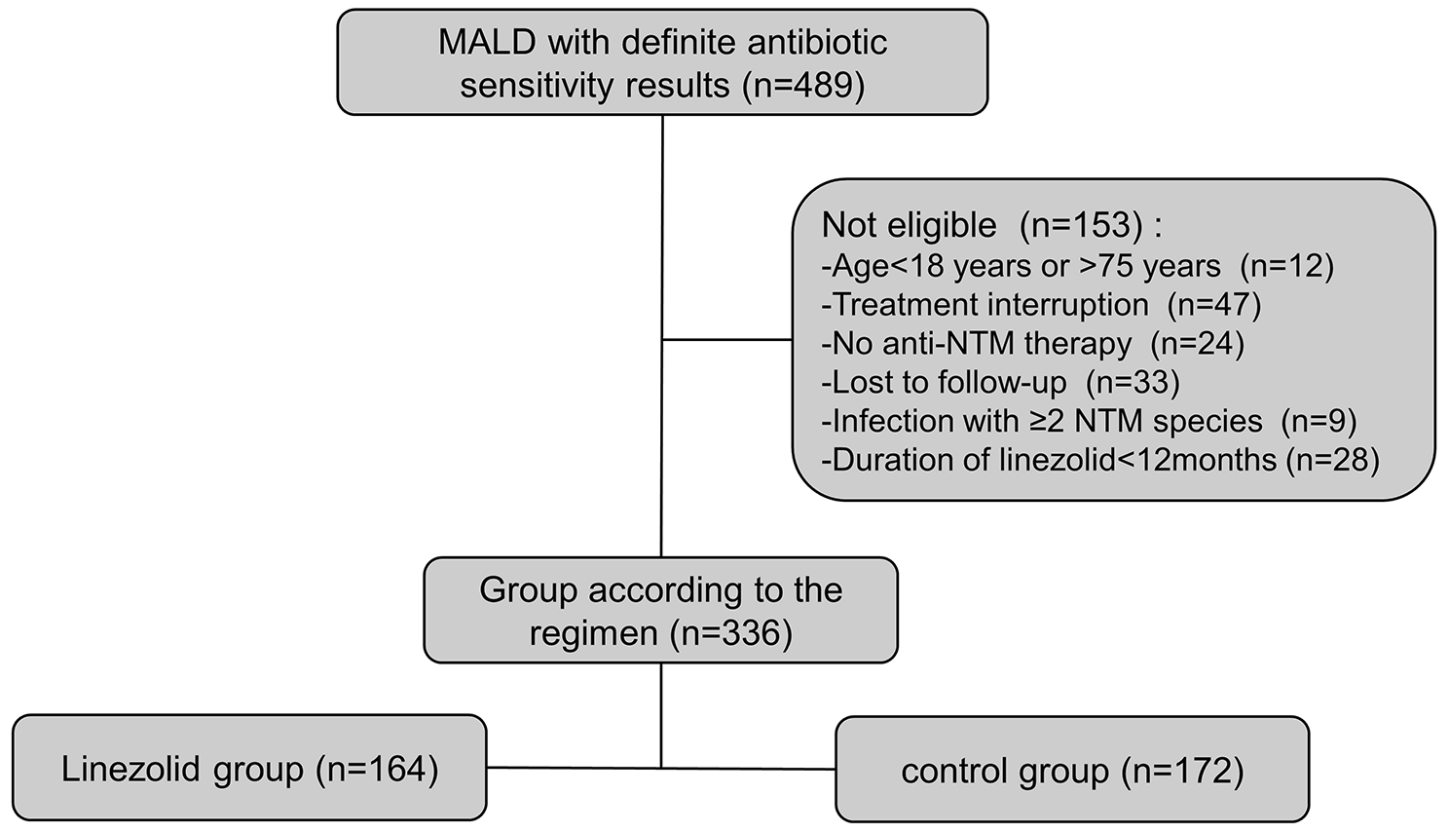
Identification and disposition of patients with *Mycobacterium abscessus* pulmonary disease (MAB-PD)

We included 336 patients in the final analysis, 164 (48.8%) in the linezolid group and 172 (51.2%) in the control group (Fig. 1; Table 1). Overall, the mean age was 55 ± 14 years (range: 21–74). The two groups had no significant differences in age, sex, BMI, symptoms (such as cough, sputum, hemoptysis, and dyspnea), medical history (NTM pulmonary disease, chronic obstructive pulmonary disease, diabetes mellitus), and NTM pulmonary disease presentation (fibro-cavitary and nodular bronchiectatic). Aside from linezolid, these two groups also had no significant differences in the use of different antibiotics (P > 0.05). However, most of these antibiotics (amikacin, clarithromycin, clofazimine, cefoxitin, and doxycycline) were used for a shorter duration (P < 0.05, Table 2). The details of the treatment regimens are in Supplementary Table 1.

**Table 1:** Baseline characteristics of the linezolid and control groups*

Variable	Linezolid group	Control group	*P* value
Number	164	172	
Age, years	54.6 ± 11.9	57.0 ± 14.7	0.153
Gender, female	112 (68.3)	104 (60.5)	0.134
BMI, kg/m^2^	17.4 ± 5.0	18.5 ± 5.7	0.158
NTM-PD, retreatment	64 (39.0)	60 (34.9)	0.432
Previous pulmonary TB	34 (20.7)	42 (24.4)	0.419
Comorbidity			
COPD	12 (7.3)	18 (10.5)	0.312
Diabetes Mellitus	8 (4.9)	6 (3.5)	0.524
Immunosuppressed	6 (3.7)	9 (5.2)	0.485
Respiratory symptoms			
Cough	122 (74.4)	139 (80.8)	0.158
Expectoration	122 (74.4)	139 (80.8)	0.158
Hemoptysis	66 (40.2)	80 (46.5)	0.247
Fever	25 (15.2)	30 (17.4)	0.586
Dyspnea	24 (14.6)	27 (15.7)	0.786
Chest CT findings			
Nodular bronchiectatic	119 (72.6)	134 (77.9)	0.256
Fibrocavitary	58 (35.3)	49 (28.5)	0.176
Other	6 (3.7)	8 (4.7)	0.649

*Numbers indicate n (%) or mean ± SD

BMI: body mass index; NTM-PD: nontuberculous mycobacterial pulmonary disease; TB: tuberculous; COPD: chronic obstructive pulmonary disease

**Table 2:** Frequency and duration of antibiotics used in the linezolid and control groups

Regimen	Number (%)		*P* value	Duration* (months)		*P* value
	Linezolid group	Control group		Linezolid group	Control group	
LZD	164 (100.0)	0 (0.0)	< 0.001	15.8(15.0,16.6)	--	--
AMK	146 (89.0)	162 (94.2)	0.087	15.6(15.2,16.5)	18.1(18.0,19.0)	< 0.01
CLR/AZM	133 (81.1)	152 (88.4)	0.063	15.9(15.0,18.5)	18.0(17.8,19.0)	< 0.01
CFZ	74 (45.1)	95(55.2)	0.064	16.5(16.1,18.0)	18.0(18.0,19.0)	< 0.01
FOX	61 (37.2)	79 (45.9)	0.104	2.6(2.3,2.6)	3.0(2.5,3.0)	< 0.01
IMP	8 (4.9)	11 (6.4)	0.547	2.3(2.2,2.3)	2.6(2.5,3.0)	0.02
DOX	20(12.2)	28 (16.3)	0.275	16.5(16.2,17.0)	17.5(17.5,18.4)	< 0.01
MFX	6 (3.7)	9 (5.2)	0.485	16.9(16.0,16.73)	18.0(17.5,19.5)	< 0.01

* Numbers indicate median (IQR)

IQR: interquartile range; LZD, linezolid; AMK, amikacin; CLR, clarithromycin; AZM, azithromycin; CFZ, Clofazimine; FOX, cefoxitin; DOX, doxycycline; IMP, imipenem; MFX, moxifloxacin; --: no statistical value

### Antibiotic susceptibilities

Table 3 shows the results of susceptibility testing. The rate of linezolid-resistant isolates was lower in the linezolid group (0.6% [1/164] vs. 92.4% [159/172]; *P <* 0.001). The two groups had no other significant differences in drug susceptibility.

**Table 3:** Antibiotic susceptibility of *M. abscessus* isolates.^*^

Drug	Total (n = 336)			Linezolid group (n = 164)			Control group (n = 172)			*χ* ^*2*^	*P* value
	S	I	R	S	I	R	S	I	R		
AMK	306 (91.1)	12 (3.6)	18 (5.3)	150 (91.5)	6 (3.6)	8 (4.9)	156 (90.7)	6 (3.5)	10 (5.8)	0.149	0.928
FOX	44 (13.1)	169 (50.3)	123 (36.6)	20 (12.2)	81 (49.4)	63 (38.4)	24 (14.0)	88 (51.2)	60 (34.8)	0.537	0.765
CIP	19 (5.6)	5 (1.5)	312 (92.9)	11 (6.7)	3 (1.8)	150 (91.5)	8 (4.6)	2 (1.2)	162 (94.2)	0.694	0.707
CLR	143 (42.6)	12 (3.6)	181 (53.8)	69 (42.1)	6 (3.7)	89 (54.2)	74 (43.0)	6 (3.5)	92 (53.5)	0.034	0.983
DOX	41 (12.2)	16 (4.8)	269 (80.0)	22 (13.4)	7 (4.3)	135 (82.3)	19 (11.1)	9 (5.2)	144 (83.7)	0.570	0.752
IMP	8 (2.4)	15 (4.5)	313 (93.1)	4 (2.4)	8 (4.9)	152 (92.7)	4 (2.3)	7 (4.1)	161 (93.6)	0.135	0.935
LZD	166 (49.4)	10 (3.0)	160 (47.6)	159 (97.0)	4 (2.4)	1 (0.6)	7 (4.1)	6 (3.5)	159 (92.4)	295.583	< 0.001
MNO	64 (19.0)	36 (10.7)	236 (70.3)	36 (22.0)	15 (9.1)	113 (68.9)	28 (16.3)	21 (12.2)	123 (71.5)	2.235	0.327
MFX	14 (4.2)	4 (1.2)	318 (94.6)	7 (4.3)	1 (0.6)	156 (95.1)	7 (4.1)	3 (1.7)	162 (94.2)	0.923	0.630
TOB	50 (14.9)	46 (13.7)	240 (71.4)	25 (15.2)	20 (12.2)	119 (72.6)	25 (14.5)	26 (15.1)	121 (70.4)	0.609	0.737
SOX	16 (4.8)	- -	320 (95.2)	8 (4.9)	- -	156 (95.1)	8 (4.7)	- -	164 (95.3)	0.01	0.922

*Numbers indicate n (%)

S, susceptible; I, intermediate; R, resistant; AMK, amikacin; FOX, cefoxitin; CIP, ciprofloxacin; CLR, clarithromycin; DOX, doxycycline; IMP, imipenem; LZD, linezolid; MNO, minocycline; MFX, moxifloxacin; TOB, tobramycin; SOX, sulfamethoxazole

### Treatment outcomes

The treatment success rate did not differ significantly between the linezolid group (56.1%, 92/164) and the control group (48.8%, 84/172) (Table 4). The median treatment duration was significantly shorter in the linezolid group (16.0 months [IQR: 15.0–17.0] vs. 18.0 months [IQR: 16.0–18.0], *P* < 0.01). Although a low BMI (≤ 18.5; aOR = 2.061, *P* < 0.05, 95%CI: 1.209–3.515) and cefoxitin use (aOR = 0.598, *P* < 0.05, 95%CI: 0.374–0.956) were associated with treatment outcome, there was no significant association of linezolid with outcome (Table 5).

**Table 4:** Treatment outcomes of the linezolid and control groups.^*^

Variable	Linezolid group (n = 164)	Control group (n = 172)	*χ* ^*2*^	*P* value
Symptom relief				
Cough	97 (79.5)	91 (65.5)	2.048	0.152
Expectoration	97 (79.5)	90 (64.7)	2.416	0.120
Fever	23 (92.0)	28 (93.3)	--	1.000^a^
Hemoptysis	45 (68.2)	52 (65.0)	0.164	0.685
Dyspnea	17 (70.8)	21 (77.8)	0.323	0.570
Treatment Success	92 (56.1)	84 (48.8)	1.774	0.183
Culture conversion	88 (53.7)	80 (46.5)	1.715	0.190
Lesion change				
Absorption	112 (68.3)	84 (48.8)	13.074	< 0.001
No change	32 (19.5)	42 (24.4)	1.177	0.278
Progression	20 (12.2)	46 (26.8)	11.258	0.001
Cavity change				
No	6 (10.3)	12 (24.5)	3.798	0.051
Yes				
Closure	32 (55.2)	14 (28.6)	7.669	0.006
Smaller	12 (20.7)	12 (24.5)	0.220	0.639
Larger or new	8 (13.8)	11 (22.4)	1.363	0.243

*Numbers indicate n (%)

^a^*P*-value corrected using Fisher’s exact method when the expectation was below 5, --: no statistical value

**Table 5:** Multivariate analysis of factors associated with treatment failure in patients with MAB-PD.

Factor	aOR	95%CI	*P* value
LZD	0.785	0.479–1.286	0.136
CFZ	0.785	0.479–1.286	0.944
AMK	0.864	0.378–1.977	0.730
CLR/AZM	0.829	0.437–1.570	0.565
FOX	0.598	0.374–0.956	0.033
DOX	0.971	0.594–1.588	0.759
IMP	0.965	0.502–1.260	0.840
MFX	1.294	0.791–2.119	0.108
Female	0.532	0.299–1.003	0.070
BMI ≤ 18.5 kg/m^2^	2.061	1.209–3.515	0.008
Age, years			
≤ 45	1(ref)		
45–65	1.285	0.813–2.574	0.356
≥65	1.430	0.799–2.559	0.138

aOR: adjusted odds ratio, 95% CI: 95% confidence interval, LZD, linezolid; AMK, amikacin; CLR, clarithromycin; AZM, azithromycin; CFZ, Clofazimine; FOX, cefoxitin; DOX, doxycycline; IMP, imipenem; MFX, moxifloxacin, BMI, body mass index

**Fig. 2:**
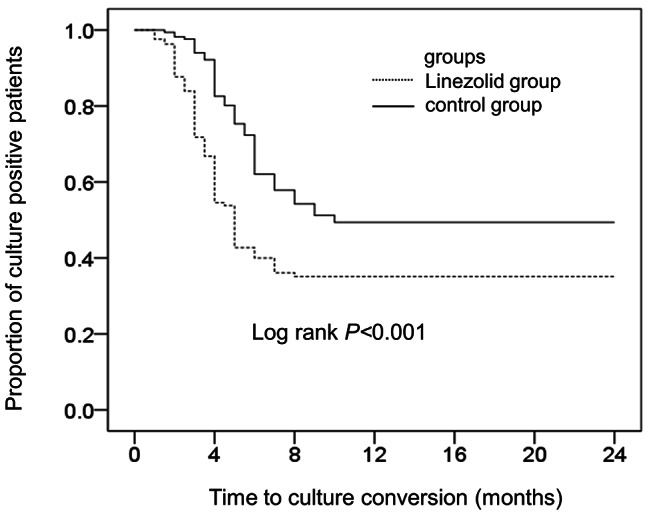
Sputum culture conversion rate in the linezolid and control groups

The rate of sputum culture conversion did not differ significantly between the linezolid group (53.7%, 88/164) and the control group (46.5%, 80/172), but the time to sputum culture conversion was significantly shorter in the linezolid group (3.5 months [IQR: 2.5–4.4] vs. 5.5 months [IQR: 4.0–6.8]; log-rank test: *P* < 0.01; Fig. 2).

**Fig. 3:**
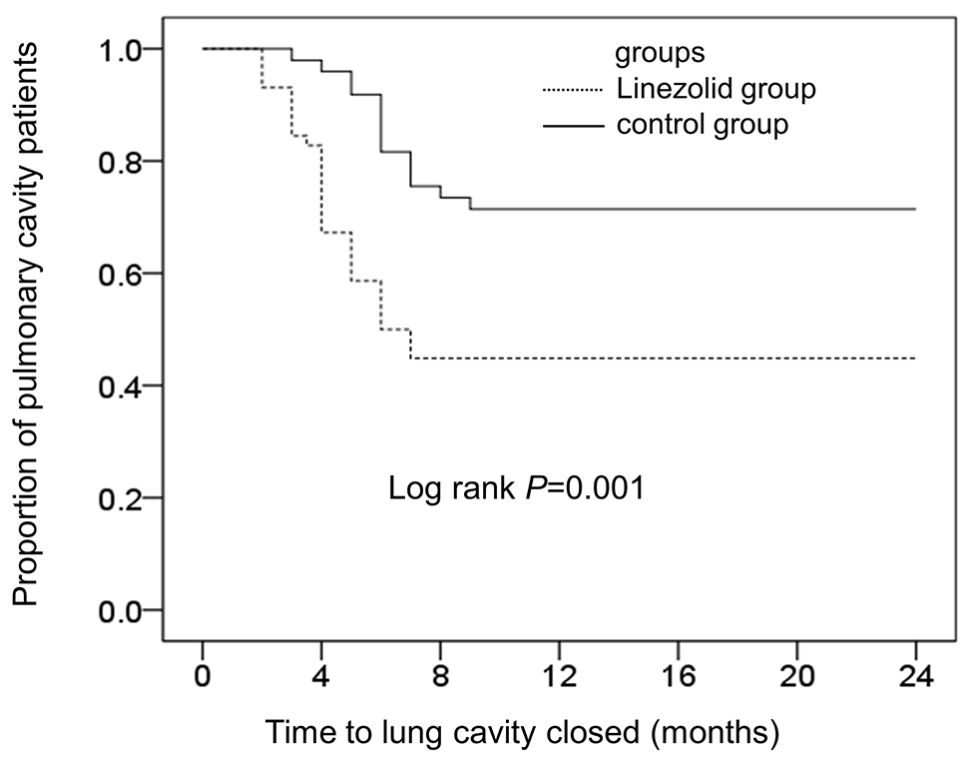
Cavity closure rate in the linezolid and control groups

**Fig. 4:**
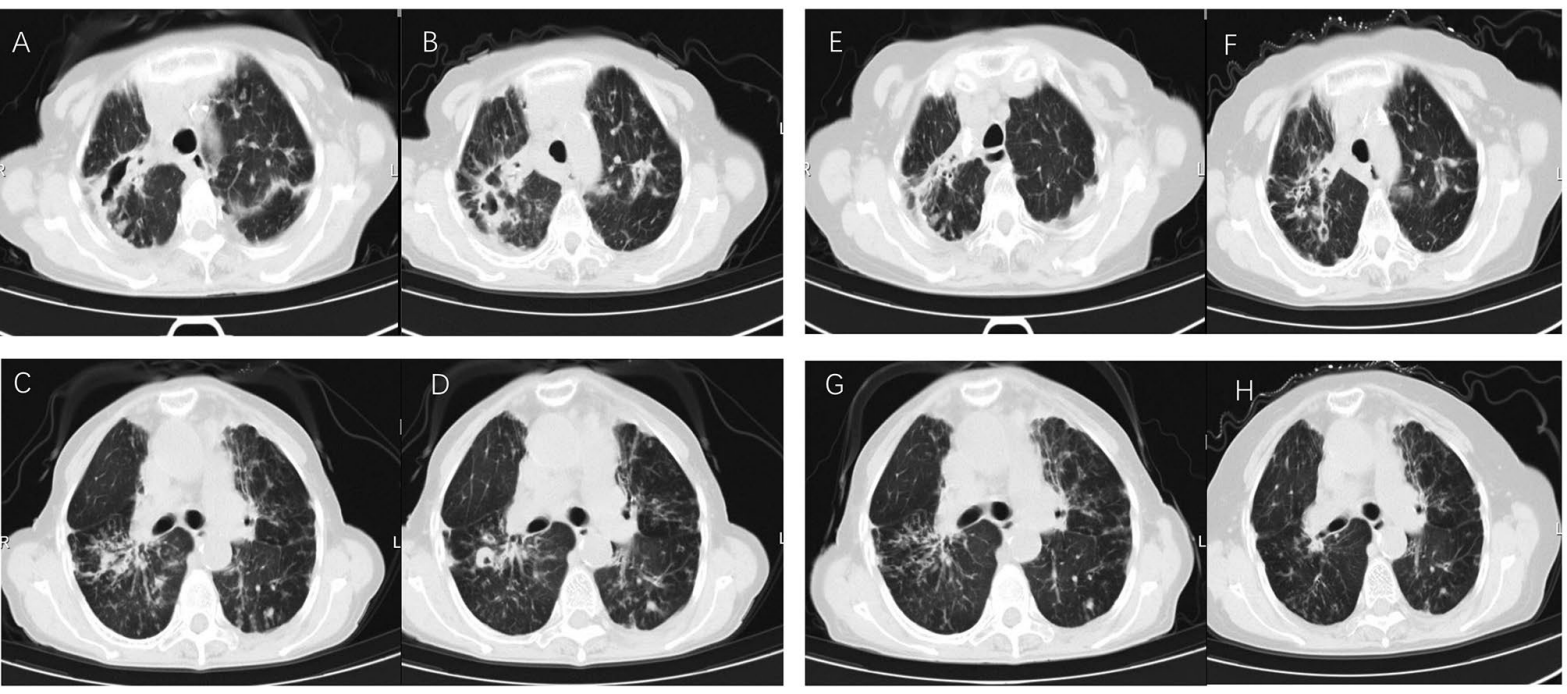
Chest CT results of a 70-year-old female who was admitted for MAB-PD. On admission, the chest CT showed multiple cavities (upper right) and patchy shadows (upper left) in A and B; there were also patchy shadows and a cavity (lower right), and bronchiectasis with patchy shadows (left) in C and D. After 1 year of treatment, the chest CT showed a smaller cavity (upper right) in E and F; there was also cavity closure and absorption of pulmonary lesions (lower) in G and H

Serial CT scans indicated improvement in 112 patients (68.3%; 112/164) in the linezolid group and 84 patients (48.8%; 84/172) in the control group. Table 4 shows that the linezolid group had a higher rate of cavity closure (55.2% [32/58] vs. 28.6% [, 14/49]; *P* < 0.01) and more rapid cavity closure (3.5 months [IQR: 2.5–4.4] vs. 5.5 months [IQR: 4.0–6.8] log-rank test: *P* < 0.01; Fig. 3). Figure 4 shows the chest CT findings of a representative female patient with MAB-PD who was treated with a linezolid-containing regimen.

### Adverse events

All adverse events were confirmed and treated based on BTS guidelines [1]. The most common adverse effects were gastrointestinal disturbances, liver injury, renal injury, bone marrow suppression (leucopenia, thrombocytopenia, and anemia), peripheral neuropathy, dermatological changes, and hearing loss.

Anemia was significantly more common in the linezolid group (17.7% [29/164] vs. 1.7% [3/172]; *P* < 0.001; Table 6). If a patient developed an episode of anemia during antibiotic treatment, appropriate symptomatic treatment was considered for mild anemia, or the dosage was decreased to 300 mg/day (moderate anemia), or usage of linezolid was halted (severe anemia). The median time from the start of linezolid to anemia was 1.6 months (IQR: 1.3–1.8). Peripheral neuropathy was significantly more common in the linezolid group (12.8% [21/164] vs. 0.6 [1/172]; *P* < 0.001), and developed after a median of 7.1 months (IQR: 3.3–9.0). In practice, if peripheral neuropathy was suspected, then nutritional neurotherapy, such as vitamin B6 and methylcobalamin, were administered in a timely manner. However, if severe peripheral neuropathy was observed, then usage of linezolid can be stopped. The two groups had no significant differences in any other adverse events.

**Table 6:** Adverse events in the linezolid and control groups^*^

Adverse event	Linezolid group (n = 164)	Control group (n = 172)	*P* value
Nausea/vomiting	20 (12.2)	23 (13.4)	0.747
Liver injury	11 (6.7)	10 (5.8)	0.735
Renal injury	3 (1.8)	4 (2.3)	1.000
Anemia	29 (17.7)	3 (1.7)	< 0.001
Thrombocytopenia	7 (4.3)	2 (1.2)	0.098
Leucopenia	4 (2.4)	2 (1.2)	0.439
Peripheral neuropathy	21 (12.8)	1 (0.6)	< 0.001
Optic neuropathy	4 (2.4)	0 (0.0)	0.056
Rash	12 (7.3)	13 (7.6)	0.933
Skin discoloration (pink to brownish-black)	73 (44.5)	91 (62.9)	0.124
Ichthyosis	30 (18.3)	44 (25.6)	0.107
Drug-induced fever	5 (3.0)	5 (2.9)	0.939
Tinnitus or hearing loss	4 (2.4)	3 (4.7)	0.718
QTc prolongation	11 (6.7)	14 (8.1)	0.617
Others	4 (2.4)	3 (1.7)	0.718

*Numbers indicate n (%)

## Discussion

*M. abscessus* is one of the most pathogenic mycobacteria, and it also has high antibiotic-resistance, making it challenging to treat MAB-PD. Because MAB-PD has a high prevalence and the causative pathogen is often drug resistant, many patients have poor treatment outcome and relapse [5, 8, 9, 16, 21]. Although most in vitro studies showed susceptibility to cefoxitin, azithromycin, and amikacin, clinical studies indicated that efficacy was unsatisfactory [22]. In addition, acquired resistance to clarithromycin has made treatment of MAB-PD even more difficult [23]. Several in vitro studies showed that linezolid was active against *M. abscessus* [16, 24], and the ATS/ERS/ESCMID/IDSA recently recommended linezolid for treatment of MAB-PD [19]. Despite this, linezolid use has been limited in clinical practice due to its high cost and its association with adverse effects. Evidence regarding the efficacy and safety of linezolid for treatment of MAB-PD is insufficient. This led us to investigate use of linezolid as a treatment for MAB-PD.

Linezolid is the first of a new class of antimicrobial agents —the oxazolidinones — that have activity against Gram-positive bacteria. Current regimens used to treat MAB-PD are limited because few antibiotics reach effective concentrations in serum and tissues. However, linezolid has good tissue penetration, making it a good choice for treatment of MAB-PD [25]. In our study, 47.0% of *M. abscessus* isolates were resistant to linezolid, similar to the findings of Ye et al. [26]. Previous studies reported that the susceptibility to linezolid varied from about 29–68% [15–17, 21]. The reported mechanisms of linezolid resistance include mutations in 23 S rRNA genes and/or ribosomal proteins L3, L4, and L22 and posttranscriptional modification of 23 S rRNAs [27–29]. A recent study of linezolid-resistant clinical isolates of *M. abscessus* found that a potential mechanism of resistance involved drug efflux pumps [26]. Although linezolid has activity against *M. abscessus* and achieves high tissue and blood concentrations, many clinical isolates are resistant, and its efficacy in the treatment of MAB-PD is questionable. Furthermore, our linezolid group had a lower rate of linezolid resistant isolates (0.6%). Due to the high rates of antibiotic resistance rate of *M. abscessus*, few drugs are available for use in clinical practice. Although susceptibility is tested in vitro and the results may differ from the actual effects in vivo, linezolid is still used for treatment of MAB-PD in patients with resistant isolates.

Treatment success was slightly higher in the linezolid group (56.1% vs. 48.8%), but the difference was not significant. In addition, our results were similar in the multivariable regression analysis. However, the linezolid group had a significantly shorter duration of treatment and a significantly shorter culture conversion time. The first clinical report of the successful use of linezolid for treatment of an *M. abscessus* infection was published in 2018 [30]. Since then, additional evidence has demonstrated that linezolid has significant clinical activity in MAB-PD. For example, one study in 2019 suggested that linezolid combined with amikacin, imipenem, and tigecycline could increase the treatment success rate [31]. Another prospective study of patients with MAB-PD [32] showed that an intensified regimen (amikacin 400–600 mg/day, azithromycin 250 mg/day, moxifloxacin 400 mg/day, linezolid for three months at 600 mg/day and then 300 mg/day, cefoxitin 8–12 g/day for 4 weeks) led to better outcome than the conventional regimen (clarithromycin 1000 mg/day or azithromycin 250 mg/day, moxifloxacin 400 mg/day, amikacin 400–600 mg/day, cefoxitin 4 g/day for 12 weeks). In particular, the rate of sputum culture conversion was much higher in patients receiving linezolid (81.3% vs. 29%), and the median time to culture conversion in the linezolid group was only 1 month (IQR: 1–1) [32]. These data differs from our findings, possibly because this previous study only included 16 patients who received linezolid. Moreover, this previous study did not report drug susceptibility of the *M. abscessus* isolates. Our data suggested that linezolid use could significantly shorten the median time to sputum culture conversion (3.5 vs. 5.5 months) and the treatment time (16.0 vs. 18.0 months). The more rapid response to the linezolid regimen may be due to better tissue penetration, a longer in vivo half-life, or that the in vivo concentration exceeded the susceptibility breakpoint for bacteria in plasma and epithelial lining fluid [33, 34].

Our study showed that use of a linezolid-containing regimen for treatment of MAB-PD improved lung status judged by CT imaging; in particular, linezolid significantly increased the rate of cumulative cavity closure (55.2% vs. 28.6%) and reduced the median time to cavity closure (4.0 vs. 6.0 months). Tang et al. reported similar findings in patients with XDR-TB [35]. Another study found that linezolid had strong bactericidal activity against mycobacteria during the logarithmic growth phase and a long-term sterilizing activity during the stationary growth phase [36].

Previous studies reported that myelosuppression was the most common adverse event associated with linezolid use [37, 38]. In agreement, our linezolid group had a significantly higher prevalence of anemia (17.7% vs. 1.7%). After treatment onset, 14 patients in our linezolid group required dosage reduction to 300 mg/day until the end of treatment, although the remaining 15 patients with anemia maintained the primary dosage of 600 mg/day. Our study also found peripheral neuropathy was another common adverse reaction associated with linezolid use, consistent with previous studies [38].

Our study has some limitations. First, to evaluate the effect of linezolid, it would be better to examine patients who received fixed regimens. However, our comparison is based on existing evidence, due to the retrospective study design of this study. Therefore, a future multicenter, randomized, prospective study is needed to affirm and validate the efficacy of linezolid for treatment of MAB-PD. Second, *M. abscessus* can be divided into subspecies, such as *M. abscessus* and *M. massiliense*. It is known that *M. abscessus* and *M. massiliense* infections have different rates of treatment response. Therefore, it would be helpful to have data regarding subspecies. Although MALDI-TOF MS is a useful tool for identification of non-tuberculous mycobacteria and can accurately discriminate *M. abscessus* and *M. massiliense*, about 70% of our patients received this examination, and the rests were tested with PCR reverse dot-blot hybridization, a method that cannot define subspecies.

## Conclusions

Although there were no significant differences in treatment success, patients who received linezolid had a shorter treatment duration, more rapid lung cavity closure, and more rapid sputum culture conversion than the control patients. However, clinicians must remain alert to anemia and peripheral neuropathy as adverse events when prescribing linezolid. We suggest that further studies evaluate additional candidate antibiotics for treatment of MAB-PD, and verify whether linezolid can be used as a core antibiotic in patients infected with susceptible isolates. We also suggest that further in vivo and in vitro studies investigate the effectiveness of additional established broad-spectrum drugs, such as tigecycline, and develop new drugs for treatment of MAB-PD.

### Electronic supplementary material

Below is the link to the electronic supplementary material.

Supplementary Material 1

## Data Availability

The datasets used and/or analyzed during the current study are available from the corresponding author on reasonable request.

## References

[1] Haworth CS, Banks J, Capstick T, Fisher AJ, Gorsuch T, Laurenson IF (2017). British Thoracic Society guidelines for the management of non-tuberculous mycobacterial pulmonary Disease (NTM-PD). Thorax.

[2] Ratnatunga CN, Lutzky VP, Kupz A, Doolan DL, Reid DW, Field M (2020). The rise of Non-tuberculosis Mycobacterial Lung Disease. Front Immunol.

[3] Mirsaeidi M, Farshidpour M, Allen MB, Ebrahimi G, Falkinham JO (2014). Highlight on advances in Nontuberculous Mycobacterial Disease in North America. Biomed Res Int.

[4] Hu C, Huang L, Cai M, Wang W, Shi X, Chen W (2019). Characterization of non-tuberculous mycobacterial pulmonary Disease in Nanjing district of China. BMC Infect Dis.

[5] Tan Y, Deng Y, Yan X, Liu F, Tan Y, Wang Q (2021). Nontuberculous mycobacterial pulmonary Disease and associated risk factors in China: a prospective surveillance study. J Infect.

[6] Cheng LP, Chen SH, Lou H, Gui XW, Shen XN, Cao J, et al. Factors Associated with Treatment Outcome in patients with Nontuberculous Mycobacterial Pulmonary Disease: a large Population-based Retrospective Cohort Study in Shanghai. Trop Med Infect Dis. 2022;7(2). 10.3390/tropicalmed7020027. 10.3390/tropicalmed7020027 PMC8876156 35202222

[7] Griffith DE, Daley CL (2022). Treatment of Mycobacterium abscessus Pulmonary Disease. Chest.

[8] Li G, Pang H, Guo Q, Huang M, Tan Y, Li C (2017). Antimicrobial susceptibility and MIC distribution of 41 Drugs against clinical isolates from China and reference strains of nontuberculous mycobacteria. Int J Antimicrob Agents.

[9] Griffith DE (2019). Mycobacterium abscessus and Antibiotic Resistance: same as it ever was. Clin Infect Dis.

[10] Kwak N, Dalcolmo MP, Daley CL, Eather G, Gayoso R, Hasegawa N, et al. Mycobacterium abscessus pulmonary Disease: individual patient data meta-analysis. Eur Respir J. 2019;54(1). 10.1183/13993003.01991-2018. 10.1183/13993003.01991-2018 30880280

[11] Pasipanodya JG, Ogbonna D, Ferro BE, Magombedze G, Srivastava S, Deshpande D, et al. Systematic review and meta-analyses of the effect of chemotherapy on pulmonary Mycobacterium abscessus outcomes and Disease recurrence. Antimicrob Agents Chemother. 2017;61(11). 10.1128/AAC.01206-17. 10.1128/AAC.01206-17 PMC5655093 28807911

[12] Falzon D, Schünemann HJ, Harausz E, González-Angulo L, Lienhardt C, Jaramillo E, et al. World Health Organization treatment guidelines for drug-resistant Tuberculosis, 2016 update. Eur Respir J. 2017;49(3). 10.1183/13993003.02308-2016. 10.1183/13993003.02308-2016 PMC5399349 28331043

[13] Cavusoglu C, Soyler I, Akinci P (2007). Activities of Linezolid against nontuberculous mycobacteria. New Microbiol.

[14] Wallace RJ, Brown-Elliott BA, Ward SC, Crist CJ, Mann LB, Wilson RW (2001). Activities of linezolid against rapidly growing mycobacteria. Antimicrob Agents Chemother.

[15] Broda A, Jebbari H, Beaton K, Mitchell S, Drobniewski F (2013). Comparative drug resistance of Mycobacterium abscessus and M. Chelonae isolates from patients with and without cystic fibrosis in the United Kingdom. J Clin Microbiol.

[16] Hatakeyama S, Ohama Y, Okazaki M, Nukui Y, Moriya K (2017). Antimicrobial susceptibility testing of rapidly growing mycobacteria isolated in Japan. BMC Infect Dis.

[17] Zhang Z, Lu J, Song Y, Pang Y (2018). In vitro activity between linezolid and other antimicrobial agents against Mycobacterium abscessus complex. Diagn Microbiol Infect Dis.

[18] Griffith DE, Aksamit T, Brown-Elliott BA, Catanzaro A, Daley C, Gordin F (2007). An official ATS/IDSA statement: diagnosis, treatment, and prevention of nontuberculous mycobacterial Diseases. Am J Respir Crit Care Med.

[19] Daley CL, Iaccarino JM, Lange C, Cambau E, Wallace RJ, Andrejak C (2020). Treatment of Nontuberculous Mycobacterial Pulmonary Disease: an Official ATS/ERS/ESCMID/IDSA Clinical Practice Guideline. Clin Infect Dis.

[20] van Ingen J, Aksamit T, Andrejak C, Böttger EC, Cambau E, Daley CL (2018). Treatment outcome definitions in nontuberculous mycobacterial pulmonary Disease: an NTM-NET consensus statement. Eur Respir J.

[21] Weng YW, Huang CK, Sy CL, Wu KS, Tsai HC, Lee SS (2020). Treatment for Mycobacterium abscessus complex-lung Disease. J Formos Med Assoc.

[22] Nie WJ, Duan HF, Huang HR, Lu Y, Bi DP, Chu NH (2014). Species identification of Mycobacterium abscessus subsp. Abscessus and Mycobacterium abscessus subsp. bolletii using rpoB and hsp65, and susceptibility testing to eight antibiotics. Int J Infect Dis.

[23] Koh WJ, Jeon K, Lee NY, Kim B-J, Kook Y-H, Lee S-H (2011). Clinical significance of differentiation of Mycobacterium massiliense from Mycobacterium abscessus. Am J Respir Crit Care Med.

[24] Shirata M, Yoshimoto Y, Marumo S, Tamai K, Fukui M (2020). In vitro efficacy of combinations of eight antimicrobial agents against Mycobacteroides Abscessus complex. Int J Infect Dis.

[25] Honeybourne D, Tobin C, Jevons G, Andrews J, Wise R (2003). Intrapulmonary penetration of linezolid. J Antimicrob Chemother.

[26] Ye M, Xu L, Zou Y, Li B, Guo Q, Zhang Y, et al. Molecular Analysis of Linezolid-Resistant Clinical isolates of Mycobacterium abscessus. Antimicrob Agents Chemother. 2019;63(2). 10.1128/AAC.01842-18. 10.1128/AAC.01842-18 PMC6355594 30478161

[27] Long KS, Vester B (2012). Resistance to linezolid caused by modifications at its binding site on the ribosome. Antimicrob Agents Chemother.

[28] Zong Z, Jing W, Shi J, Wen S, Zhang T, Huo F, et al. Comparison of in Vitro Activity and MIC distributions between the Novel Oxazolidinone Delpazolid and Linezolid against Multidrug-resistant and extensively drug-resistant Mycobacterium tuberculosis in China. Antimicrob Agents Chemother. 2018;62(8). 10.1128/AAC.00165-18. 10.1128/AAC.00165-18 PMC6105784 29844043

[29] Tian Y, Li T, Zhu Y, Wang B, Zou X, Li M (2014). Mechanisms of linezolid resistance in staphylococci and enterococci isolated from two teaching hospitals in Shanghai, China. BMC Microbiol.

[30] Inoue T, Tsunoda A, Nishimoto E, Nishida K, Komatsubara Y, Onoe R (2018). Successful use of linezolid for refractory Mycobacterium abcessus Infection: a case report. Respir Med Case Rep.

[31] Chen JH, Zhao L, Mao YH, Ye MP, Guo Q, Zhang YJ (2019). Clinical efficacy and adverse effects of Antibiotics used to treat Mycobacterium abscessus Pulmonary Disease. Front Microbiol.

[32] Li H, Tong L, Wang J, Liang Q, Zhang Y, Chu N et al. An Intensified Regimen Containing Linezolid Could Improve Treatment Response in Mycobacterium abscessus Lung Disease. *Biomed Res Int* 2019; 2019: 8631563. 10.1155/2019/8631563. 10.1155/2019/8631563 PMC6885786 31828137

[33] Boselli E, Breilh D, Rimmelé T, Djabarouti S, Toutain J, Chassard D (2005). Pharmacokinetics and intrapulmonary concentrations of linezolid administered to critically ill patients with ventilator-associated Pneumonia. Crit Care Med.

[34] Conte JE, Golden JA, Kipps J, Zurlinden E (2002). Intrapulmonary pharmacokinetics of linezolid. Antimicrob Agents Chemother.

[35] Tang S, Yao L, Hao X, Zhang X, Liu G, Liu X (2015). Efficacy, safety and tolerability of linezolid for the treatment of XDR-TB: a study in China. Eur Respir J.

[36] Koh WJ, Kwon OJ, Gwak H, Chung JW, Cho SN, Kim WS (2009). Daily 300 mg dose of linezolid for the treatment of intractable multidrug-resistant and extensively drug-resistant Tuberculosis. J Antimicrob Chemother.

[37] Poon YK, La Hoz RM, Hynan LS, Sanders J, Monogue ML (2021). Tedizolid vs Linezolid for the Treatment of Nontuberculous Mycobacteria Infections in Solid Organ Transplant recipients. Open Forum Infect Dis.

[38] Winthrop KL, Ku JH, Marras TK, Griffith DE, Daley CL, Olivier KN (2015). The tolerability of linezolid in the treatment of nontuberculous mycobacterial Disease. Eur Respir J.

