# Supplementary material for: Effectiveness and safety of regimens containing linezolid for treatment of Mycobacterium abscessus pulmonary Disease
Source: Ann Clin Microbiol Antimicrob. 2023 Dec 6;22:106. doi: 10.1186/s12941-023-00655-2 (PMC10702010; doi:10.1186/s12941-023-00655-2)
Supplement: Supplementary file 1 — Supplementary Material 1 [file 12941_2023_655_MOESM1_ESM.docx]

**Supplementary Table 1. Anti-mycobacterium treatment regimens in the two groups**

| **Group** | **Initial phase** | | | **Continuation phase** | | |
| --- | --- | --- | --- | --- | --- | --- |
|  | **Regimen** | **n** | **Duration***  **months** | **Regimen** | **n** | **Duration***  **months** |
| Linezolid group | AMK(IV)+CLR/AZM+LZD | 47 | 2.4(1.5-3.5) | AMK(N)+CLR/AZM+CFZ+ LZD | 20 | 13.5(12.0-15.5) |
|  | AMK(IV)+CLR/AZM+CFZ +LZD | 27 | 2.5(1.9-3.2) | AMK(N)+CLR/AZM+LZD | 70 | 13.0(13.8-17.6) |
|  | AMK(IV)+DOX+CLR/AZM+ LZD | 8 | 3.0(2.0-3.3) | AMK(N)+CFZ+LZD | 22 | 14.3(13.5-19.1) |
|  | AMK(IV)+DOX+CFZ+LZD | 12 | 2.5(2.0-3.5) | AMK(N)+DOX+LZD | 7 | 14.0(13.8-16.6) |
|  | AMK(IV)+FOX+CLR/AZM+LZD | 24 | 2.6(2.4-3.2) | AMK(N)+DOX+CLR/AZM +LZD | 7 | 13.5(13.2-18.0) |
|  | AMK(IV)+FOX+CFZ+LZD | 19 | 2.6(1.4-3.5) | CLR/AZM+CFZ+LZD | 19 | 15.5(13.6-20.4) |
|  | AMK(IV)+IMP+CFZ+LZD | 2 | 2.1 | CLR/AZM+LZD | 12 | 14.0(13.6-18.4) |
|  | AMK(IV)+IMP+CLR/AZM +LZD | 6 | 2.3(1.9-3.0) | DOX+CLR/AZM+LZD | 1 | 14.7 |
|  | AMK(IV)+MFX+CFZ+LZD | 1 | 2.7 | MFX+CLR/AZM+LZD | 5 | 14.0(13.3-18.1) |
|  | FOX+CLR/AZM+CFZ+LZD | 13 | 2.6(1.2-3.5) | MFX+CFZ+LZD | 1 | 15.2 |
|  | FOX+MFX+CLR/AZM +LZD | 5 | 2.5(1.6-3.4) |  |  |  |
| Control group | AMK(IV)+CLR/AZM+CFZ | 62 | 2.6(1.9-4.2) | AMK(N)+CLR/AZM | 45 | 16.0(14.0-18.5) |
|  | AMK(IV)+CLR/AZM+DOX | 15 | 2.5(1.3-3.5) | AMK(N)+CLR/AZM+CFZ | 74 | 15.5(13.5-19.0) |
|  | AMK(IV)+FOX+CFZ | 15 | 2.1(1.3-3.2) | AMK(N)+CLR/AZM+DOX | 21 | 15.7(13.9-18.5) |
|  | AMK(IV)+FOX+CLR/AZM+ CFZ | 12 | 2.5(1.5-3.5) | AMK(N)+CFZ | 15 | 16.5(14.5-18.3) |
|  | AMK(IV)+FOX+CLR/AZM | 40 | 3.0(2.0-3.6) | AMK(N)+MFX+DOX | 4 | 16.7(14.0-18.2) |
|  | AMK(IV)+FOX+DOX+CLR/AZM | 6 | 2.7(1.8-3.8) | CLR/AZM+DOX | 2 | 17.5 |
|  | AMK(IV)+IMP+CLR/AZM | 5 | 2.5(1.5-3.0) | CLR/AZM+CFZ | 6 | 16.6(16.0-19.1) |
|  | AMK(IV)+IMP+CFZ+CLR/AZM | 2 | 2.6 | MFX+CLR/AZM | 4 | 17.0(15.5-19.5) |
|  | AMK(IV)+MFX+DOX | 5 | 3.0(2.0-3.5) | MFX+CFZ | 1 | 17.2 |
|  | FOX+CLR/AZM+CFZ | 4 | 3.0(1.6-3.5) |  |  |  |
|  | FOX+MFX+CLR/AZM | 2 | 2.8 |  |  |  |
|  | IMP+DOX+CLR/AZM | 2 | 2.5 |  |  |  |
|  | IMP+MFX+CLR/AZM | 2 | 1.9 |  |  |  |

*Numbers indicate median (IQR).

IQR: interquartile range; IV: intravenous; N: nebulised; AMK: amikacin; FOX: cefoxitin; CLR: clarithromycin; AZM: azithromycin; CFZ: clofazimine; LZD: linezolid; DOX: doxycycline; IMP: imipenem; MFX: moxifloxacin.
